# Facile Synthesis of Gold Nanoparticles with Alginate and Its Catalytic Activity for Reduction of 4-Nitrophenol and H_2_O_2_ Detection

**DOI:** 10.3390/ma10050557

**Published:** 2017-05-19

**Authors:** Xihui Zhao, Zichao Li, Yujia Deng, Zhihui Zhao, Xiaowen Li, Yanzhi Xia

**Affiliations:** 1 School of Chemistry and Chemical Engineering, Qingdao University, Qingdao 266071, China; dengyujia@qdu.edu.cn (Y.D.); zzh@qdu.edu.cn (Z.Z.); xiaowenli@qdu.edu.cn (X.L.); 2 Collaborative Innovation Center for Marine Biomass Fibers, Materials and Textiles of Shandong Province, Institute of Marine Bionbased Materials, Qingdao University, Qingdao 266071, China; 3 State Key Laboratory Breeding Base of New Fiber Materials and Modern Textile, School of Materials Science and Engineering, Qingdao University, Qingdao 266071, China; zichaoli@qdu.edu.cn

**Keywords:** gold nanoparticles, alginate, catalytic activity

## Abstract

Gold nanoparticles (AuNPs) were synthesized using a facile solvothermal method with alginate sodium as both reductant and stabilizer. Formation of AuNPs was confirmed by UV-vis spectroscopic analysis. The synthesized AuNPs showed a localized surface plasmon resonance at approximately 520–560 nm. The AuNPs were characterized using transmission electron microscopy, X-ray diffraction and dynamic light scattering. Transmission electron microscopy revealed that the AuNPs were mostly nanometer-sized spherical particles. Powder X-ray diffraction analysis proved the formation of face-centered cubic structure of Au. Catalytic reduction of 4-nitrophenol was monitored via spectrophotometry using AuNPs as catalyst, and further a non-enzymatic sensor was fabricated. The results demonstrated that AuNPs presented excellent catalytic activity and provided a sensitive response to H_2_O_2_ detection.

## 1. Introduction

Gold nanoparticles (AuNPs) have recently received intensive interest because of their unique physicochemical properties and their various potential chemical applications [1,2,3]. However, despite these advantages, AuNPs synthesis usually involves chemical reduction reaction in the presence of various reducing agents and capping agents, in which toxic chemicals are used [4,5,6]. Therefore, development of an efficient, green, and eco-friendly method to prepare AuNPs is a worthy endeavor. Studies have reported that many natural compounds, such as those derived from fungi, algae, bacteria, and plants, were used in green synthesis of AuNPs [7,8,9,10]. These natural compounds-mediated procedures for synthesis of AuNPs represent advantages over conventional chemical and physical methods, as they are simply, low-cost, energy-efficient, and nontoxic green routes [11,12,13,14]. In this regard, we focused our attention on natural polysaccharide extracted from seaweed, which is a proven source of bioactive compounds [15]. Furthermore, natural polysaccharides can be used for AuNPs synthesis in a different way [16]. Alginate, one kind of polysaccharide, isolated from marine algae, is a biocompatible, non-toxic, and biodegradable compound. Alginate is a copolymer of β-d-mannuronic acid (M) and α-l-guluronic acid (G) and has a number of free carboxyl and hydroxyl groups distributed along its backbone [17,18]. In the preparation of the AuNPs, the hydroxyl groups of alginate can reduce the Au (III) ions to Au (0). Meanwhile, the interaction between Au and functional groups such as hydroxyl and carboxyl groups prevented AuNPs from aggregation and provided smaller AuNPs. Recently, several researchers reported the synthesis of AuNPs with alginate [19,20,21]. Nevertheless, few studies report the applications of AuNPs-algiante in catalytic reduction of 4-nitrophenol (4-NP) and sensor preparation for detecting H_2_O_2_.

The present work reports on AuNPs synthesis via a facile solvothermal method with alginate as both reducing and stabilizing agent. Furthermore, this work discusses the application of these newly synthesized AuNPs in catalytic reduction of 4-nitrophenol (4-NP). 4-NP is one of the most common hazardous organic pollutants and contaminates the environment. While, the reduced product, 4-nitroaniline (4-AP) is a very important intermediate for the production various drugs, dyeing agent, photographic developers, and corrosion inhibitors [22,23]. Nonetheless, the reduction of 4-NP by traditional methods are mostly ineffective due to the high stability and low solubility of 4-NP in water. Therefore, direct catalytic reduction of 4-NP into 4-AP using a facile and efficient method is indispensable in the present day context.

Additionally, most previous studies indicate that hydrogen peroxide (H_2_O_2_) is an important intermediate species in food, pharmaceutical, industrial, and environmental analysis, but, bioaccumulation of H_2_O_2_ produces oxidative stress and results in severe damage to cells [24,25]. Nowadays, considerable attention has been focused on rapid, low-cost, accurate determination of H_2_O_2_ in trace levels in biological and various water samples [26,27,28]. Many analytical methods have been developed to detect H_2_O_2_, such as chemiluminescence, spectrophotometry, titrimetry and electrochemical method. Among them, non-enzymatic electrochemical sensors are attracted much attention due to its advantages of low-cost, simplicity, high sensitivity and high selectivity [29,30,31]. The standard reduction potential for the reaction of H_2_O_2_ is 1.77 V, and AuNPs, as a typical noble metal NPs, has been reported to exhibit good catalytic activity toward the reduction of H_2_O_2_ [32]. Unfortunately, a great number of AuNPs tend to easily aggregate and change shape due to the poor balance between the nucleation and growth processes and the high surface energy, which results in a remarkable decrease in their catalytic and electrochemical activity and selectivity undesirably. To overcome these obstacles, in this study, AuNPs were stabled and dispersed by alginate. non-enzymatic H_2_O_2_ sensor was fabricated with the as-prepared alginate-AuNPs and electrochemical property of the sensor was investigated.

## 2. Experimental Section

Sodium alginate (SA) was supplied by Jiejing Seaweed Co. Ltd., Rizhao, Shandong Province, China. Analytical grade chloroauric acid (HAuCl_4_), 4-nitrophenol (4-NP) and sodium borohydride (NaBH_4_) were supplied by Beijing Chemical Factory (Beijing, China) and used as supplied without further purification. All solutions were prepared with deionized water.

In a typical preparation, sodium alginate was dissolved in distilled water. After alginate was completely dissolved, certain amount of chloroauric acid (HAuCl_4_, 24 mM) precursor was added drop-wise into 5 mL of sodium alginate solution. The reaction mixture was subsequently heated to facilitate the reduction of gold ions.

UV-vis spectra of AuNPs in alginate solution were obtained on a Shimadzu UV3150. Transmission electron microscopy (TEM) images were obtained by a JEM-1200EX microscope at an accelerating voltage of 100.0 kV. X-ray diffraction (XRD) measurements were performed on a powder X-ray diffractometer (D/MAX-RB) using CuKα radiation (λ = 0.15418 nm) over a 2θ range of 5°–80°. Particle size distribution and zeta potential was measured by Differential Light Scattering (DLS) Malvern Zetasizer Nano ZS (Malvern Instruments Ltd., Worcestershire, UK).

We chose the reduction of 4-NP by NaBH_4_ as a model reaction to evaluate the catalytic activity of the prepared AuNPs. First, 4.5 mL of freshly prepared 0.05 M NaBH_4_ aqueous solution was mixed with 0.5 mL of 1 mM 4-NP aqueous solution. Subsequently, 20 μL of alginate-AuNPs solution was added into the mixture. The conversion of 4-NP into 4-AP at room temperature was monitored by a UV-vis spectrophotometer (Shimadzu UV3150, Kyoto, Japan).

Electrochemical measurements were performed at room temperature with a conventional three-electrode system controlled by a CHI 760E electrochemical workstation (Shanghai CH Instrument Co. Ltd., Shanghai, China). A platinum coil and a saturated calomel electrode (SCE) were used as the counter and reference electrodes, respectively. The glassy carbon electrode (GCE) was used as working electrode, which was surface modified with the prepared materials. The GCE (4.0 mm in diameter) was firstly polished with 0.3 and 0.05 mm alumina slurries on a polishing cloth and then rinsed with deionized water, followed by ultrasonic treatment in deionized water and ethanol successively. Au suspension (5 μL), which was prepared using 1.0 mM HAuCl_4_, was dropcasted on the cleaned GCE and dried in air at room temperature. The modified electrodes were identified as AuNPs/GCE.

**Scheme 1:**
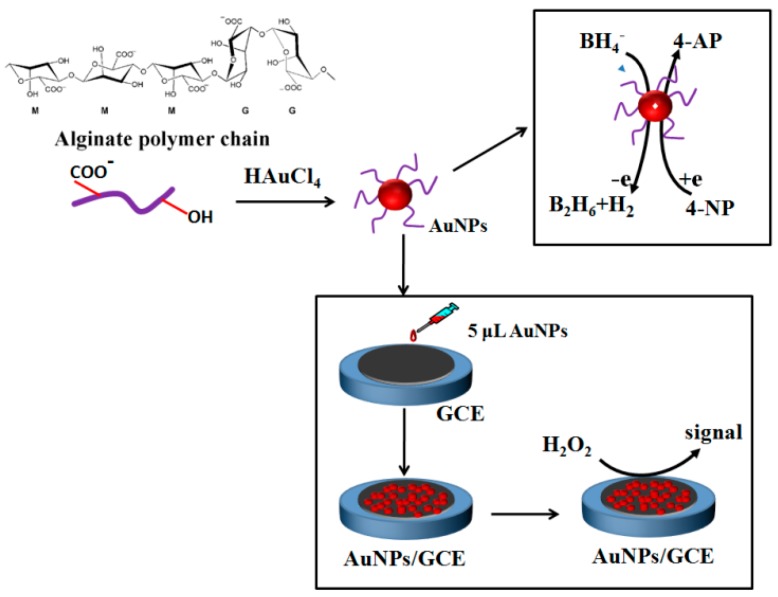
Schematic illustration of the formation of AuNPs with alginate and its application for the catalytic hydrogenation of 4-NP to 4-AP and the non-enzymatic H_2_O_2_ sensor.

Scheme 1 shows a schematic diagram of the formation of AuNPs with alginate, as well as the catalytic hydrogenation of 4-NP to 4-AP and the fabrication of the non-enzymatic H_2_O_2_ sensor.

## 3. Results and Discussion

### 3.1. Effect of Reaction Parameters on the Synthesis of Gold Nanoparticles

**Figure 1:**
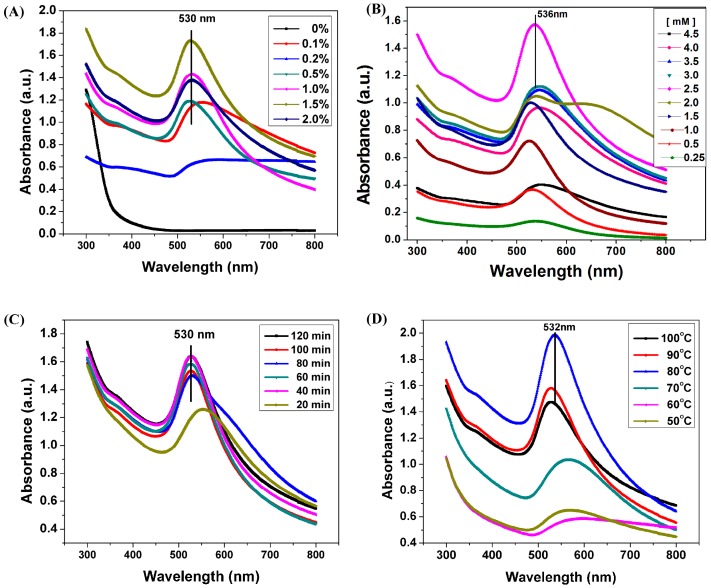
Effect of reaction parameters on the synthesis of gold nanoparticles. (**A**) UV-vis spectra of AuNPs prepared using different concentrations of sodium alginate in the presence of HAuCl_4_ (2.5 mM) at 90 °C for 60 min; (**B**) UV-vis spectra of AuNPs prepared using different concentrations of HAuCl_4_, sodium alginate (1.0%) at 90 °C for 60 min; (**C**) UV-vis spectra of AuNPs prepared at different reaction time with sodium alginate 1.0%, HAuCl_4_ 1.2 mM at 90 °C; (**D**) UV-vis spectra of AuNPs prepared with different reaction temperature with sodium alginate 1.0%, HAuCl_4_ 1.2 mM, 60 min.

During heating, the color of sodium alginate solutions containing HAuCl_4_ changed slowly from yellow to pink resulting from the reduction of Au^3+^ into Au^0^ (Figure S1) [33]. UV-vis spectroscopic analysis was used to measure AuNPs formation. Figure 1A shows the UV-vis spectra of the AuNPs obtained using different concentrations (0.1–2%) of sodium alginate as reducing and stabilizing agent. The spectra showed a unique localized surface plasmon resonance (LSPR) absorption band at approximately 520–550 nm, which is associated with AuNPs formation. This result indicated that Au^3+^ could be reduced into Au^0^ by alginate due to the presence of hydroxyl (OH) groups in the polymer chain [34]. The absorption intensity increased rapidly with increased sodium alginate concentration, and this phenomenon could be attributed to the high amount of OH groups in alginate; these OH groups facilitate the reduction of Au^3+^, increasing AuNPs yield. The peak shifted toward shorter wavelength from 550 nm to 530 nm at increased alginate concentrations, indicating that the nanoparticles were well dispersed in the reactive system resulting from the stabilizing effect of alginate. Sardar et al. reported that at higher polymer concentration the initial rate of formation of AuNPs was slower and the reduction took a longer time to complete. Alginate probably formed a micelle around Au^0^ nuclei, as a result, alginate could control the growth of particles [35,36].

Preparation of AuNPs using 1.0% sodium alginate was evaluated under varying HAuCl_4_ concentrations, and the UV-vis spectra of the AuNPs obtained are shown in Figure 1B. A significant LSPR absorbance peak was centered at approximately 530 nm. With increasing Au atomic ratio, the LSPR band of AuNPs enhanced first and then decreased. This phenomenon may be attributed to change in size of the AuNPs as revealed in their TEM image. With increasing Au content, the AuNPs probably reunited and enlarged, causing the visible light absorbance to decrease, the broadening of the peak may results from increasing the polydispersity of AuNPs [36,37].

The gold nanoparticles formation was examined at different time intervals. With the passage of time from 20 to 40 min, the intensity of absorbance peaks increased and broadness of absorbance peaks decreased as shown in Figure 1C, reflecting the formation of more AuNPs. While, further prolonging the reaction duration up to 60 min, the intensity of plasmon absorption band displayed a slight decrease, also, the maximum of the absorbance peak was nearly the same, implying the as-prepared aqueous dispersions of AuNPs were very stable against aggregation. Figure 1D showed the UV–vis absorption spectroscopy of AuNPs prepared at different temperatures using 1.0% sodium alginate and 1.2 mM HAuCl_4_. It was clear from the data that the temperature played the important role to the reduction reaction and particle size, when the reaction temperature was 50 °C–70 °C, the intensity of the plasmon band was weak and very broaden around 570 nm, which indicated that the reduction efficiency was not very good and no complete transformation of Au^3+^ into gold nanopartricles was achieved at this temperature. Raising the reaction temperature up to 80 °C, the absorption band at 526 nm becomes stronger and narrower which means higher conversion of Au^3+^ to Au^0^ with smaller nanoparticles size. This might result from the reduction of Au ions by alginate molecules at higher temperature [35]. As Wang et al. reported, fast nucleation yielded smaller particles and higher particle concentration at higher temperature [38]. On the other hand, there was significant enhancement in the absorption band by rising the temperature up to 100 °C with concomitant wavelength shifted towards larger wavelength band (535 nm), indicating that larger nanosized gold were formed [39]. The possible reason is the degradation of alginate at higher temperature, resulting in the higher reducing capacity and lower stabilizing power to AuNPs.

### 3.2. TEM, XRD and DLS Analysis

**Figure 2:**
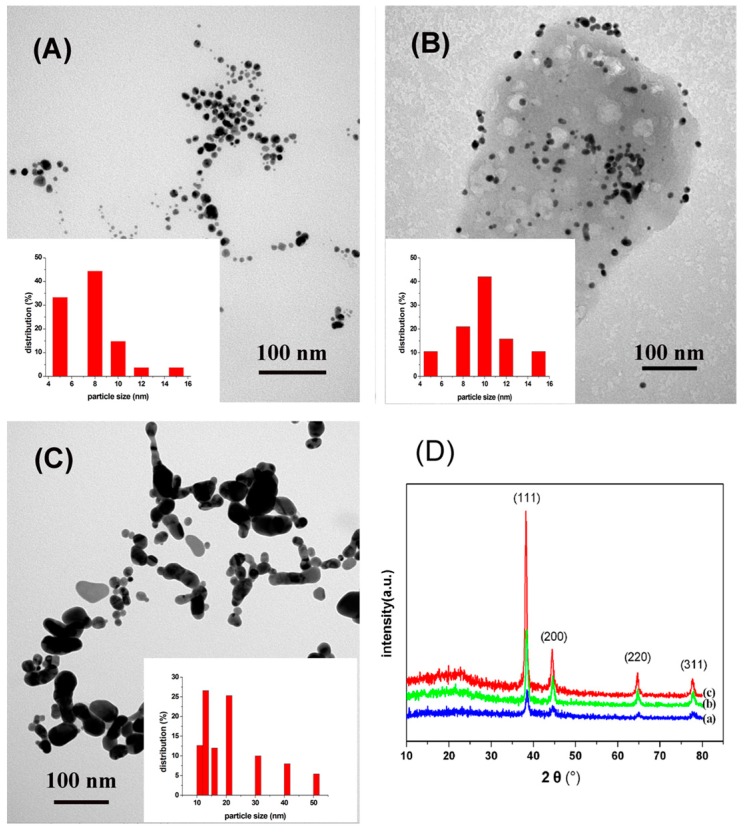
(**A**–**C**) TEM images of AuNPs prepared using 0.25, 1.0, 3.5 mM of HAuCl_4_, respectively; (**D**) XRD patterns of AuNPs prepared using different HAuCl_4_ concentrations of 0.25 (**a**); 1.0 (**b**); 3.5 (**c**) mM, respectively. The inset is the corresponding particle size analysis histogram of the gold nanoparticles.

The TEM images (Figure 2A–C) provided information on the size, morphology and dispersion of the obtained AuNPs under different concentrations of HAuCl_4_. The AuNPs mainly assumed a nearly spherical shape at lowHAuCl_4_ concentration. Additionally, the size of AuNPs varied under different concentrations of Au^3+^ ions. The histograms (the inset of Figure 2A–C) clearly illustrated that the prepared particle size was around 10 nm at low concentrations of HAuCl_4_. While, the formed AuNPs tended to aggregate when the concentration of HAuCl_4_ was increased up to 3.5 mM, large particles were found and the NPs varied from 10 to 60 nm in size. The crystalline nature of the prepared AuNPs was confirmed by wide-angle XRD analysis, and the result was shown in Figure 2D. Several distinct diffraction peaks at approximately 38.2°, 44.4°, 64.6°, and 77.6° were assigned to the reflections from the (1 1 1), (2 0 0), (2 2 0), and (3 1 1) planes of Au crystal, respectively; these peaks corroborate the crystalline structure of AuNPs and further on the basis that they can be indexed as face-centered-cubic (FCC) structure of Au [40]. Among the corresponding planes, (1 1 1) plane exhibited a higher intensity than the other planes, suggesting that the (1 1 1) plane is the predominant orientation. The intensity of the diffraction peaks varies with increasing concentration of HAuCl_4_.

**Figure 3:**
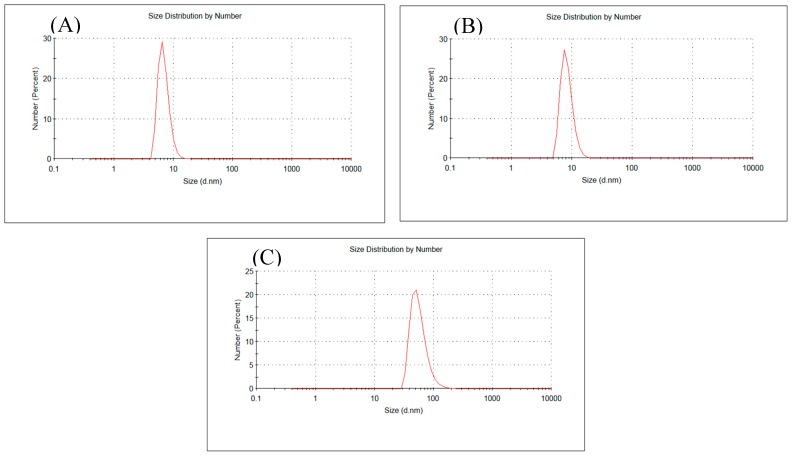
(**A**–**C**) DLS profile: Size distribution by number of AuNPs prepared using 0.25, 1.0, 3.5 mM HAuCl_4_, respectively.

**Figure 4:**
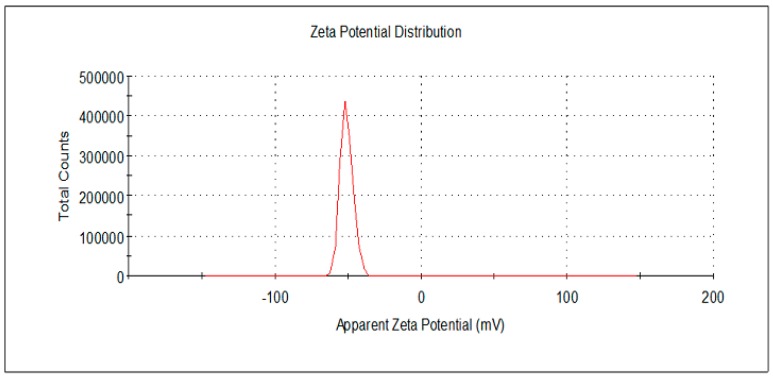
Zeta potential analysis of AuNPs prepared using 0.25 mM HAuCl_4_.

Dynamic light scattering (DLS) analysis showed the size distribution by number of particles obtained under different concentrations of HAuCl_4_ (Figure 3). The average particle size determined by DLS method was slightly increased from 7 nm to 9 nm at lower HAuCl_4_ concentration (Figure 3A,B). While, the particle size increased to be 50 nm with the increase of HAuCl_4_ concentration up to 3.5 mM. The result is in agreement with the result obtained from TEM. Additionally, a stable dispersion of particles was evident from the zeta potential of −52.3 mV (Figure 4); a zeta potential higher than 30 mV or lesser than −30 mV indicates a stable system [3,41]. Additionally, the stabilization of the AuNPs is not only due to electrostatic phenomenon, but also the polymeric structure of alginate [38,42]. Alginate can act as surface active molecules to stabilize the nanoparticles due to the extensive number of hydroxyl and carboxylic groups on alginate chain. However, further studies are required to elucidate the mechanism of biological AuNPs synthesis.

### 3.3. Catalytic Activity Study of Gold Nanoparticles

**Figure 5:**
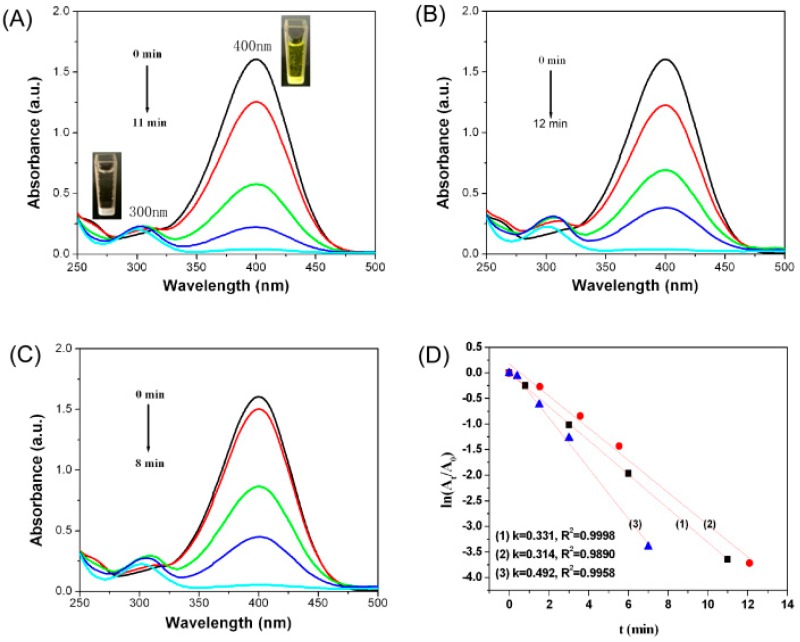
(**A**–**C**) Time-dependent absorption spectra of the reaction solution of 4-NP into 4-AP over AuNPs prepared using 0.25, 1.0, and 3.5 mM HAuCl_4_, respectively; (**D**) (1), (2), and (3) show the plot of ln(A_t_/A_0_) against the reaction time over AuNPs prepared using 0.25, 1.0, and 3.5 mM HAuCl_4_, respectively.

This work investigated the catalytic activity of AuNPs in reduction of 4-NP into 4-AP in the presence of NaBH_4_. Reduction of 4-NP by NaBH_4_ is thermodynamically feasible involving a standard reduction potential of −0.76 for 4-NP/4-AP and −1.33 V for H_3_BO_3_/BH_4_^−^ versus that of a normal hydrogen electrode; however, this reaction is kinetically restricted in the absence of a catalyst [43]. The color of 4-NP solution changed from light yellow to bright yellow immediately upon addition of NaBH_4_ solution, and the absorption peak of 4-NP shifted from 317 nm to 400 nm, which was due to the formation of 4-nitrophenolate ions under highly basic conditions (Figure S2). The absorbance of 4-NP at 400 nm decreased only slightly in the absence of a catalyst, suggesting that 4-NP was not effectively reduced by NaBH_4_ or that the reduction rate was very slow (Figure S3). By contrast, after adding AuNPs into the reaction medium, the absorption peak of 4-NP at 400 nm decreased abruptly, along with the appearance of a new absorption peak of 4-AP at 300 nm (Figure 5A–C), indicating the successful reduction of 4-NP into 4-AP. Moreover, the AuNP catalyst exhibited an efficient catalytic activity within 12 min to nearly the completion of the reaction. AuNPs act as electron relay center and initiate shifting of electron from the donor BH_4_^−^ to the acceptor 4-NP, causing reduction of 4-NP. The reactant molecules (BH_4_^−^ ion and 4-NP) were simultaneously adsorbed onto the surface of NPs, as a result, electrons transferred from BH_4_^−^ ion into 4-NP through the NPs [44].

Reduction of 4-NP fits well a pseudo-first order kinetics equation when the amount of NaBH_4_ in the reaction medium is excessive compared with that of 4-NP. Thus, ln(A_t_/A_0_) = −kt, where A_t_/A_0_ is the ratio of the absorbance at 400 nm of 4-NP at time t to that at time 0, k is the rate constant of the reaction, and t is the reaction time (min). The value of k could be determined from the slopes between ln(A_t_/A_0_) versus t. The plots of ln(A_t_/A_0_) versus time for the reduction of 4-NP by NaBH_4_ with varied forms of AuNPs as catalysts are shown in Figure 5D. Obviously, a good linear relationship between ln(A_t_/A_0_) versus time was observed, confirming pseudo-first-order kinetics. The corresponding rate constants k are 0.331, 0.314, and 0.492 min when AuNPs prepared with 0.25 (a), 1.0 (b), 3.5 (c) mM HAuCl_4_ were used, respectively. A study has shown that k is related to the total surface area of AuNPs, which depends on the size and content of AuNPs [45]. A considerably increased amount of AuNPs that formed at high HAuCl_4_ concentration will increase the catalytic activity, whereas, the increased particle size will reduce the catalytic activity.

### 3.4. Catalytic Activity Study of Gold Nanoparticles

To testify the sensing application of as-prepared AuNPs, an enzymeless H_2_O_2_ sensor has been constructed by direct deposition of the AuNPs aqueous dispersion on a bare GCE surface. The electrocatalytic activity of AuNPs modified electrodes toward H_2_O_2_ oxidation was studied using typical cyclic voltammetry (CV). According to the literature [30,46], the mechanism of H_2_O_2_ electroreduction can be summarized as the following sequence:

H_2_O_2_ + e^−^ ↔ OH_(ads)_ + OH^−^

OH_(ads)_ + e^−^ ↔ OH^−^

2OH^−.^+ H^+^ ↔ 2H_2_O

Importantly, when AuNPs was introduced in the reaction, it became more irreversible. The possible reaction may be obeyed the following equations [26,30]:

H_2_O_2_ →1/2 O_2_ + H_2_O O_2_ + 2e^−^ + 2H^+^ → H_2_O_2_ (1)

The electrocatalytic detection of H_2_O_2_ should be attributed to the reduction of H_2_O_2_ on the AuNPs surface. The oxygen generated in the reaction was turned into the detection signal at the electrode.

**Figure 6:**
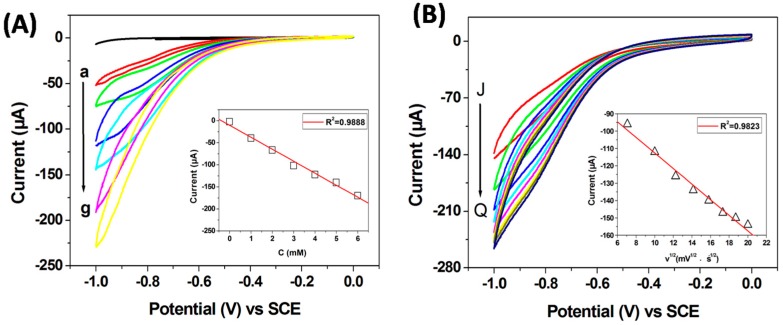
(**A**) CV curves of AuNPs/GCE obtained in N_2_-saturated 0.1 M PBS (pH 7.2) with different H_2_O_2_ concentration (from a to g: 0, 1, 2, 3, 4, 5 and 6 mM) at a 50 mV S^−1^ scan rate, the inset is the linear fitting program of the reduction peak current (0.9 V) versus the H_2_O_2_ concentration; (**B**) CV curves of AuNPs/GCE obtained in N_2_-saturated 0.1 M PBS (pH 7.2) containing 3 mM H_2_O_2_ concentration with different scan rate (from J to Q: 50, 100, 150, 200, 250, 300, 350 and 400 mV S^−1^), the inset is the linear fitting program of the reduction peak current (0.9 V) versus the square root of scan rate.

The catalytic responses of the AuNPs modified GCE by changing the concentration of H_2_O_2_ in N_2_-saturated 0.1 M PBS solution (pH 7.2) at scan rate of 50 mV s^−1^ were recorded in Figure 6A. It can be seen that only a featureless CV profile was obtained when no H_2_O_2_ was introduced in the system. After adding H_2_O_2_, the reduction current of H_2_O_2_ increased gradually with the increase of concentration in the range of 0–6 mM, which suggests that the material is electrochemically sensitive to the concentration of H_2_O_2_ [47]. The inset of Figure 6A displays a good linear relationship between the peak current and H_2_O_2_ concentration from 0 to 6 mM (R^2^ = 0.9888). To explore the reaction kinetics of H_2_O_2_ reduction on the AuNPs, the corresponding CV curves of the AuNPs/GCE scanned at different scan rates in N_2_-saturated 0.1 M PBS solution with 3 mM H_2_O_2_ are shown in Figure 6B. It can be seen the reduction current increases gradually with the scan rate increased from 50 to 400 mV s^−^^1^. As shown in the inset of Figure 6B, the peak current shows a linear increase to the square root of scan rate, indicating that the reduction reaction of H_2_O_2_ is a diffusion-controlled process [48].

## 4. Conclusions

AuNPs were synthesized using sodium alginate via a facile and green method, wherein alginate served as reducing and stabilizing agent. Sodium alginate and HAuCl_4_ concentrations, as well as reaction time and temperature exerted obvious effects on AuNPs formation. The synthesized NPs showed pronounced catalytic activity in reduction of 4-NP into 4-APin the presence of NaBH_4_. The non-enzymatic H_2_O_2_ sensor based on Au/GCE exhibited excellent sensing performances for non-enzymatic H_2_O_2_ detection.

## Supplementary Materials

The following are available online at www.mdpi.com/1996-1944/10/5/557/s1.
