# Supplementary material for: Facile Synthesis of Gold Nanoparticles with Alginate and Its Catalytic Activity for Reduction of 4-Nitrophenol and H2O2 Detection
Source: Materials (Basel). 2017 May 19;10(5):557. doi: 10.3390/ma10050557 (PMC5459079; doi:10.3390/ma10050557)
Supplement: Supplementary file 1 [file materials-10-00557-s001.pdf]

Supplementary Information

Facile Synthesis of Gold Nanoparticles with Alginate and Its Catalytic Activity for Reduction of 4-Nitrophenol and H_2O_2 Detection

Xihui Zhao[1,2,*], Zichao Li [3], Yujia Deng [1], Zhihui Zhao[1,2], Xiaowen Li[1] and Yanzhi Xia [2,3,*]

1 School of Chemistry and Chemical Engineering, Qingdao University, Qingdao 266071, China; dengyujia@qdu.edu.cn (Y.D.); zzh@qdu.edu.cn (Z.Z.); xiaowenli@qdu.edu.cn (X.L.)
2 Collaborative Innovation Center for Marine Biomass Fibers, Materials and Textiles of Shandong Province, Institute of Marine Bionbased Materials, Qingdao University, Qingdao 266071, China
3 State Key Laboratory Breeding Base of New Fiber Materials and Modern Textile, School of Materials Science and Engineering, Qingdao University, Qingdao 266071, China; zichaoli@qdu.edu.cn
* Correspondence: zhaoxihui@qdu.edu.cn (X.Z.); xiayzh@qdu.edu.cn (Y.X.); Tel.: +86-532-8595-0705 (X.Z.)

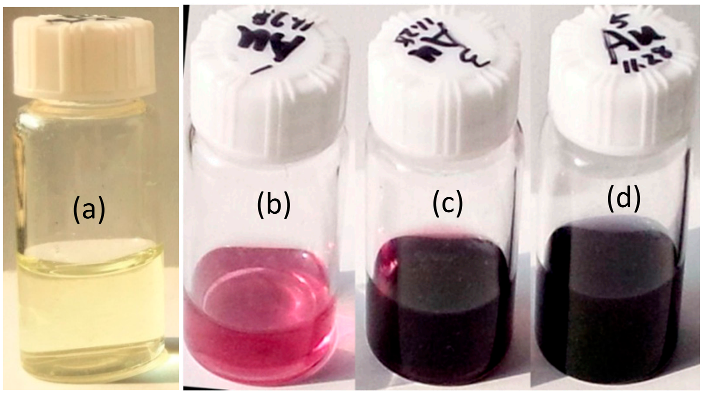

Figure S1. Digital photograph of sodium alginate solution (1.0%) mixed HAuCl4 before reaction (**a**) and after reaction 60 min at 90 °C with 0.25, 1.0, 3.5 mM of $HAuCl_4$ 50, respectively (**b-c**).

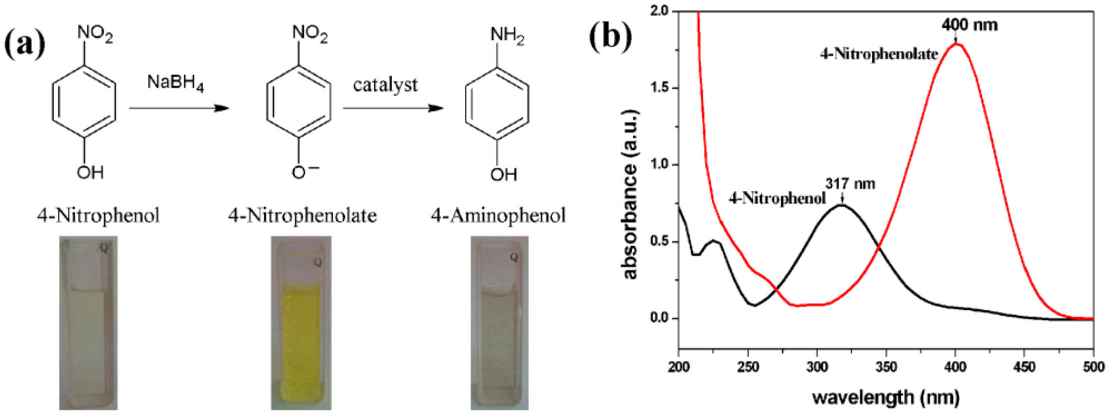

Figure S2. (**a**) The reduction reaction for the conversion of 4-NP to 4-AP; (**b**) UV-vis absorption spectra of 4-NP before and after addition of $NaBH_4$.

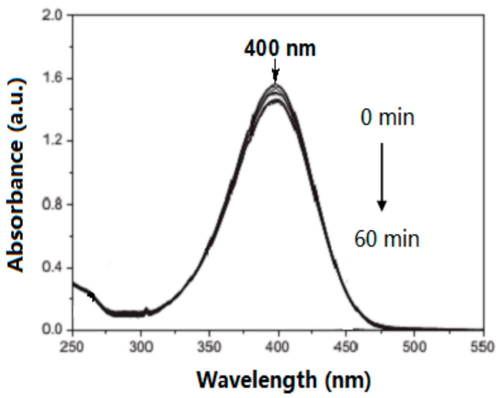

Figure S3. Time-dependent absorption spectra of the reaction solution of 4-NP to 4-AP in absence of catalyst.
